# Supplementary figures and images for: Comparison of IAA and amino acid profiles of the selected rootstocks and their accumulation in grafted scion of Cucumis sativus L
Source: PeerJ. 2025 Oct 15;13:e20159. doi: 10.7717/peerj.20159 (PMC12535229; doi:10.7717/peerj.20159)

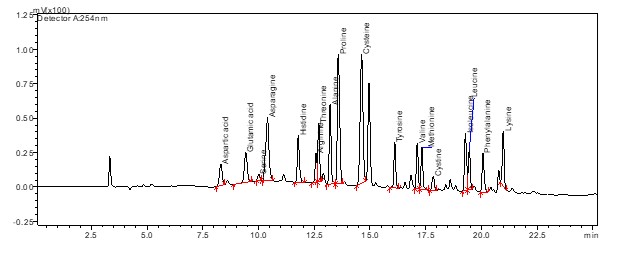

Supplement: Supplemental Information 1 [file peerj-13-20159-s001.jpg]

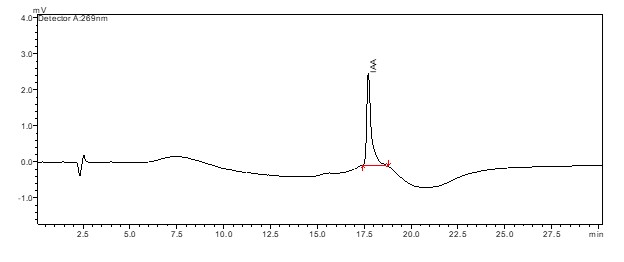

Supplement: Supplemental Information 2 [file peerj-13-20159-s002.jpg]

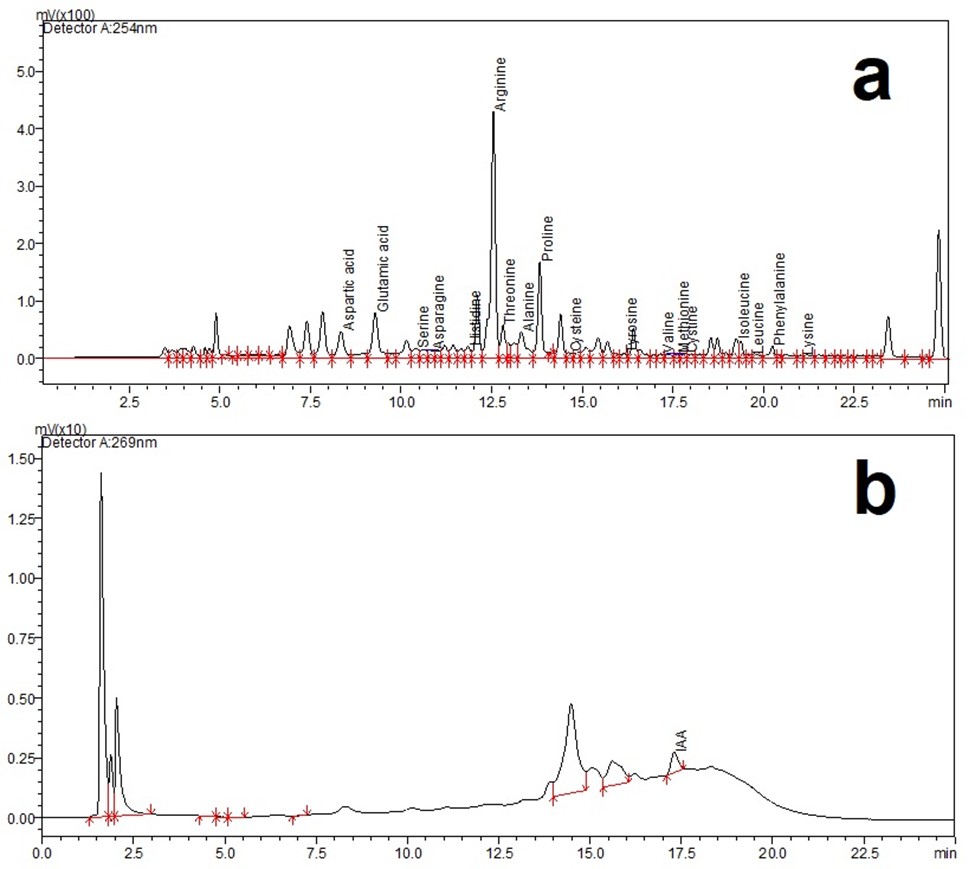

Supplement: Supplemental Information 3 [file peerj-13-20159-s003.jpg]

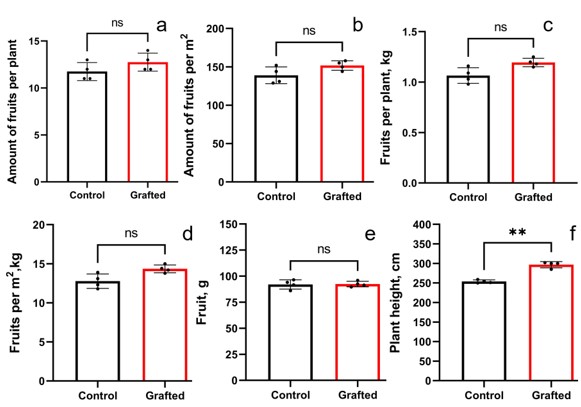

Supplement: Supplemental Information 4 — (A) Fruit per plant (B) Fruits per m 2 (C) kg per plant (D) kg per m 2 (E) Length of plants (F) Plant height. Statistically significant results between the control and grafted variants at **p < 0.01. [file peerj-13-20159-s004.jpg]

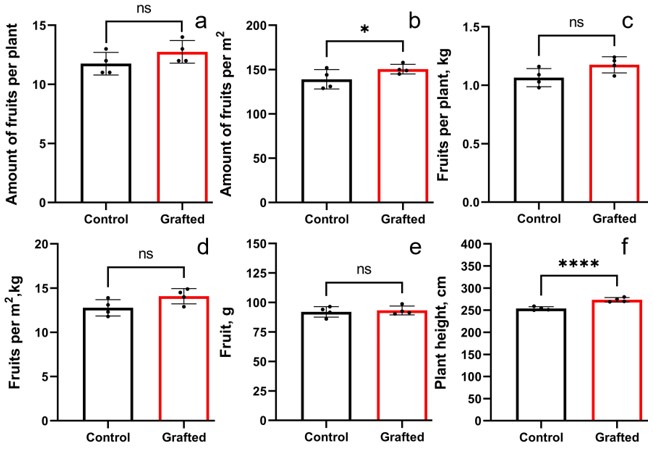

Supplement: Supplemental Information 5 — (A) Fruit per plant (B) Fruits per m 2 (C) kg per plant D) kg per m 2 (E) Length of plants (F) Plant height. Statistically significant results between the control and grafted variants at *p < 0.05, ****p < 0.0001. [file peerj-13-20159-s005.jpg]
