# Supplementary material for: Comparison of IAA and amino acid profiles of the selected rootstocks and their accumulation in grafted scion of Cucumis sativus L
Source: PeerJ. 2025 Oct 15;13:e20159. doi: 10.7717/peerj.20159 (PMC12535229; doi:10.7717/peerj.20159)
Supplement: Supplemental Information 6 [file peerj-13-20159-s006.docx]

**Supplemental files**

**Validation amino acid determination method**

Calibration curves for all analyzed amino acids were constructed using standard solutions over the concentration range of 1–100 µg/ml. As shown in Table S1, the peak area of each amino acid standard was plotted against its concentration, and linearity parameters were obtained through linear regression analysis. The determination coefficients (R²) were ≥0.995 for all AA compounds, indicating excellent linearity.

The limits of detection (LOD) and limits of quantification (LOQ) were evaluated by stepwise dilution of the standard solutions. The resulting LOD values ranged from 0.03 µg/mL to 0.16 µg/mL, while LOQ values ranged from 0.10 µg/mL to 0.47 µg/mL for all amino acids.

The accuracy and precision of the amino acid quantification method were confirmed by repeated analysis of a prepared standard mixture.

The chromatogram and peak profiles of this mixture are shown in Figure S1.

Table S1 – Retention times, linear calibration parameters, LOD and LOQ values for selected amino acids.

| No. | Amino acid | Retention time | R^2^ | Calibration Equation | Calibration Range (µg/mL) | LOD (ug/mL) | LOQ (ug/mL) |
| --- | --- | --- | --- | --- | --- | --- | --- |
| 1 | Aspartic acid | 8.387 | 0.9996 | y = 1429.4x - 1421.1 | 1–100 | 0.06 | 0.19 |
| 2 | Glutamic acid | 9.486 | 0.9998 | y = 2015.3x - 1076.4 | 1–100 | 0.03 | 0.10 |
| 3 | Serine | 10.417 | 0.9979 | y = 3084.3x - 9055.9 | 1–100 | 0.15 | 0.46 |
| 4 | Asparagine | 10.427 | 0.9977 | y = 1816.8x + 4740.6 | 1–100 | 0.16 | 0.47 |
| 5 | Histidine | 11.803 | 0.9997 | y = 2305.8x - 2791.1 | 1–100 | 0.06 | 0.17 |
| 6 | Arginine | 12.596 | 0.9997 | y = 1170.1x - 1494.1 | 1–100 | 0.06 | 0.18 |
| 7 | Threonine | 12.712 | 0.9998 | y = 2538.2x - 3107.3 | 1–100 | 0.04 | 0.13 |
| 8 | Alanine | 13.224 | 0.9989 | y = 3568.1x + 4727 | 1–100 | 0.05 | 0.14 |
| 9 | Proline | 13.589 | 0.9995 | y = 7214.1x + 1226.3 | 1–100 | 0.07 | 0.21 |
| 10 | Cysteine | 14.640 | 0.9997 | y = 8793.4x + 1234.3 | 1–100 | 0.04 | 0.11 |
| 11 | Tyrosine | 16.117 | 0.9997 | y = 1904.6x + 1495.3 | 1–100 | 0.10 | 0.32 |
| 12 | Valine | 17.124 | 0.9991 | y = 1858.6x + 2927.6 | 1–100 | 0.06 | 0.17 |
| 13 | Methionine | 17.331 | 0.9985 | y = 1812.4x + 294.6 | 1–100 | 0.13 | 0.38 |
| 14 | Cystine | 17.842 | 0.9995 | y = 783.1x + 1292.6 | 1–100 | 0.07 | 0.22 |
| 15 | Isoleucine | 19.284 | 0.9995 | y = 3035.1x + 2905.7 | 1–100 | 0.07 | 0.22 |
| 16 | Leucine | 19.463 | 0.9985 | y = 1717.1x + 447.11 | 1–100 | 0.13 | 0.39 |
| 17 | Phenylalanine | 20.076 | 0.9996 | y = 1852.5x + 2184.7 | 1–100 | 0.06 | 0.20 |
| 18 | Lysine | 20.983 | 0.9996 | y = 2401.7x + 3659.9 | 1–100 | 0.07 | 0.20 |

LOD – Limit of Detection; LOQ – Limit of Quantification.

**Validation indole-3-acetic acid determination method**

The analytical method for quantification of indole-3-acetic acid was validated in accordance with standard guidelines, including parameters such as linearity, LOD, and LOQ (Table S2). The calibration curve for IAA exhibited excellent linearity over the concentration range of 0.01–1 µg/mL, with a correlation coefficient (R²) of 0.9998. The linear regression equation obtained was y = 2945.9x – 945.1, indicating a strong proportional relationship between concentration and signal response. The LOD and LOQ were determined based on the signal-to-noise ratio approach, using S/N ratios of 3 and 10, respectively. The LOD was found to be 0.001 µg/mL, while the LOQ was 0.0019 µg/mL, confirming the high sensitivity of the method. The chromatogram and peak profiles of IAA are provided in Figure S2.

Table S2 Retention times, linear calibration parameters, LOD and LOQ values for IAA.

| No. | Standard Compound | Retention Time (min) | R^2^ | Calibration Equation | Calibration Range (µg/mL) | LOD (µg/mL) | LOQ (µg/mL) |
| --- | --- | --- | --- | --- | --- | --- | --- |
| 1 | IAA | 17.71 | 0.9998 | y = 2945.9x - 945.1 | 0.01–1 | 0.001 | 0.0019 |

LOD – Limit of Detection; LOQ – Limit of Quantification.

The chromatographic peaks of amino acids and IAA quantification of Сucurbitaceae family are provided in Figure S3.
